# Ureterocele Associated with Renal Agenesia Presented as a Pelvic Mass in an Adult

**DOI:** 10.5812/iranjradiol.10262

**Published:** 2012-12-27

**Authors:** Mohammad Ghasem Mohseni, Seyed Reza Hosseini, Alborz Salavati, Shahrzad Dadgari

**Affiliations:** 1 Department of Urology, Faculty of Medicine, Tehran University of Medical Sciences, Tehran, Iran; 2 JaameJam Radiology Center, Tehran, Iran

**Keywords:** Pelvic Region, Renal Agenesis, Ureterocele

## Abstract

Adult ureteroceles are generally known as simple ureteroceles with minimal obstructive effects 1 that can usually be managed endoscopically. Such pathology presented with acute abdominal pain and fever in a 32-year-old man with left renal agenesia, a cranial blind left ureter and left obstructed ureterocele. The retained secretions were suppurative.

## 1. Introduction

Adult ureteroceles generally known as simple ureteroceles with minimal obstructive effects are usually related to single systems They are intravesical and less obstructive and endoscopic interventions are proven effective for relieving symptoms (1). As stated in the latest edition of Campbel-Walch urology, they are four times more prevalent among females. The most accurate diagnostic modality is MRI, but they are often diagnosed by prenatal ultrasonography. As stated by the same reference, renal hypodysplasia may be accompanied by ectopic ureters, but the simple adult type ureterocele with concomitant renal agenesia is a really rare entity and only three cases have been mentioned in the literature (2, 3).

## 2. Case Presentation

**Figure 1:**
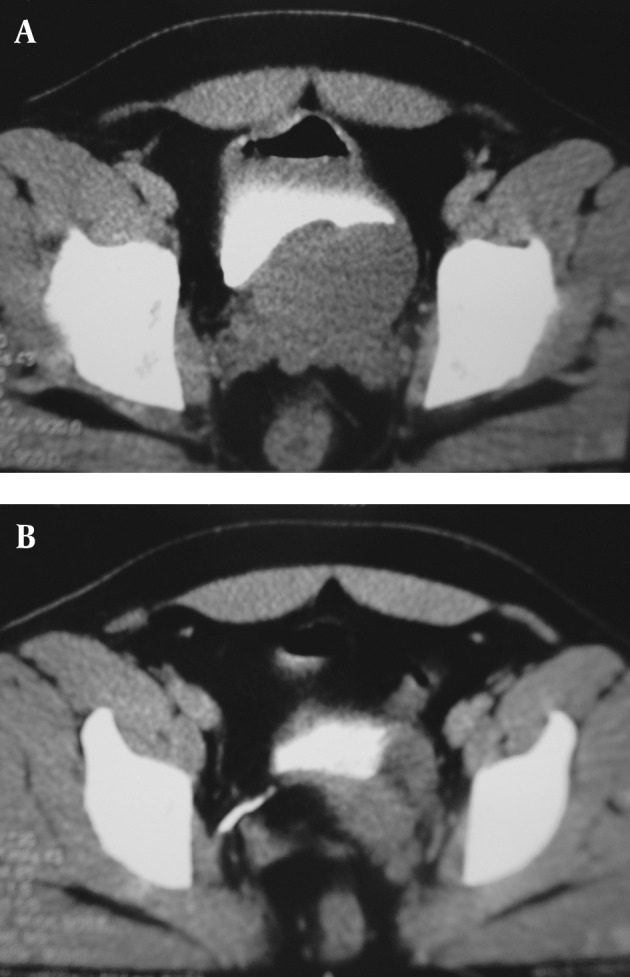
A & B, A 32-year-old man with ureterocele and renal agenesia. There is an external mass effect on the left latero-posterior side of the bladder in CT-cystogram and a normal bladder mucosa.

A 32-year-old man presented with sustained ongoing left lower quadrant (LLQ) pain aggravating three days before admission and fever. He had no gastrointestinal complaints apart from decreased appetite. Severe pain made walking impossible. He did not report any dysuria or obstructive lower urinary tract symptoms. On examination, there was LLQ tenderness without rebound or guarding, no costovertebral angle tenderness, a normal external genitalia and a low grade fever. Laboratory exam results were normal, urine analysis had one to two WBCs and one to two RBCs in the high power field (HPF). Before being referred to the hospital, he underwent abdominopelvic ultrasonography which revealed a normal right kidney, no free fluids and a normal looking spleen and liver. The left kidney could not be seen and there was a 55 × 65 mm2 solid-cystic mass postero-lateral to the left bladder wall. Abdominopelvic contrast enhanced CT did not show any stones in the urinary tract, there was left renal agenesis and a solid-cystic non enhancing pelvic mass behind the bladder (Figure 1).Office based cystoscopy revealed a normal urethra and bladder mucosa and right ureter, left ureteral orifice looked normal, but could not be cannulized and the mass effect from an extravesical source was eminent on the left lateral wall and prostatic urethra.

**Figure 2:**
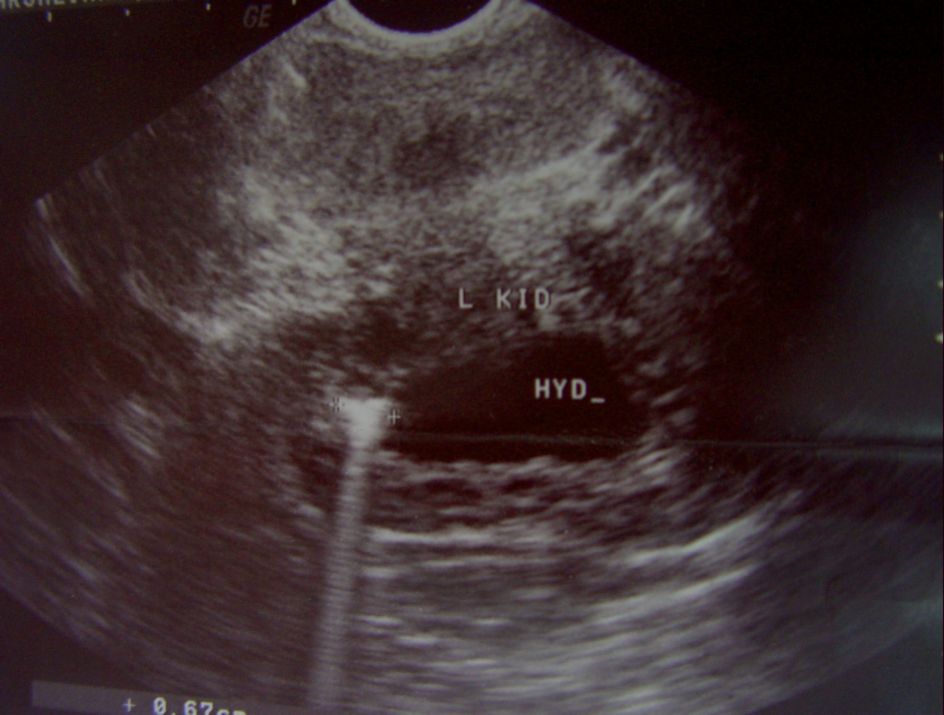
Transrectal ultrasonogram of a left side pelvic mass behind the bladder. Note the thick wall of the cystic shaped mass and the echogenic particle inside with the posterior shadow.

**Figure 3:**
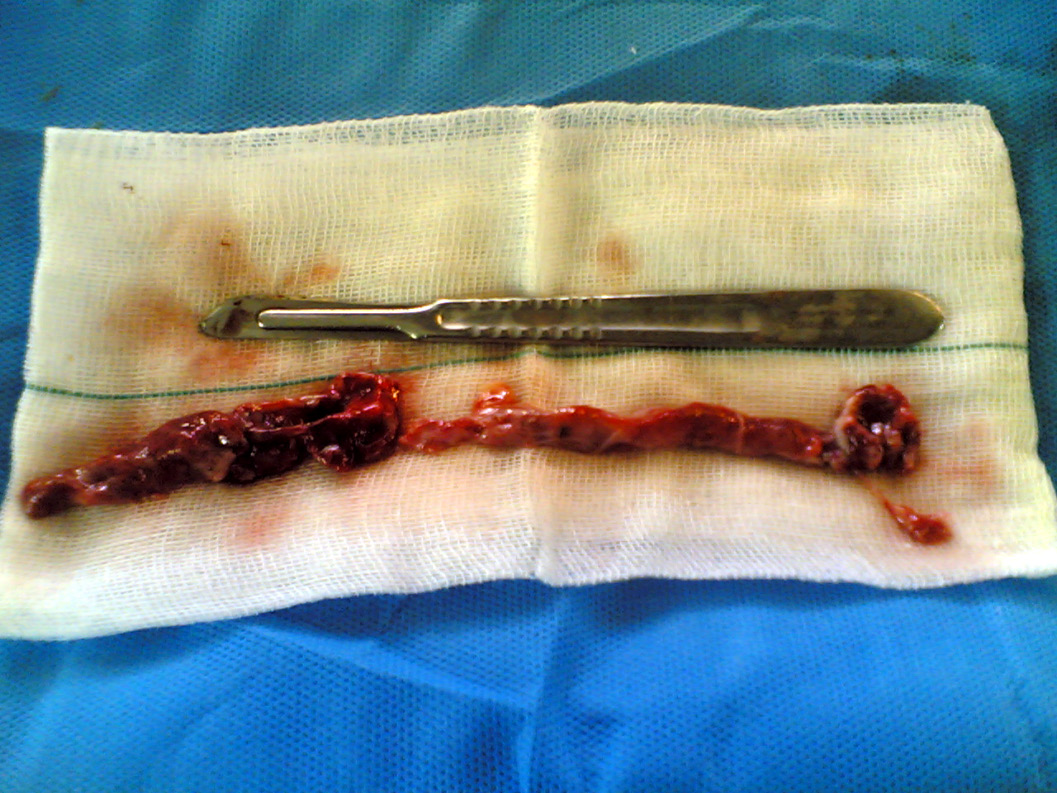
Surgical sample consisting of blind end ureter and ureterocele

On admission, a transrectal sonogram revealed a normal prostate and bilateral seminal vesicles and a 56 × 62 mm2 sized cystic mass with significant wall thickness containing fluid and an ectopic hydronephrotic pelvic left kidney was inferred (Figure 2). The DMSA renal isotope scan documented single right functional kidney in the abdominopelvic cavity. The patient underwent exploratory laparoscopy. A retrovesical pyo-ureterocele was encountered along the cranial blind end left ureter. Surgery converted to open ureterectomy and resection of the pyo-ureterocele. The pathology report confirmed dilated inflamed ureter with active suppurative inflammation (Figure 3). One month after surgery, the patient had no complaint.

## 3. Discussion

Adult orthotopic ureterocele is a well-known entity, nevertheless concomitant blind ureteral bud, renal agenesis and ureterocele have only been reported in three cases previously, and a full-blown inflammatory and septic presenting feature of this case has led to a diagnostic challenge. Diverse differential diagnoses of a pelvic mass in the male which include prostatic cysts, mullerian duct remnants and malignancies of the seminal vesicles besides the scarcity of such cases in daily practice misled both the radiologists and the urologist. The patient was operated with a preoperative diagnosis of pelvic dysplastic kidney. Maas and colleagues reported the same cases being diagnosed by pelvic MRI (2) and another similar case was reported from Spain (3). A German patient was reported by Berg et al. (4). This patient was examined for prostatic tumor in young age and on MRI, it turned out to be a seminal vesicle cyst alongside a ureterocele accompanied by ipsilateral renal agenesis (4).
